# European clinical guidelines for Tourette syndrome and other tic disorders: summary statement

**DOI:** 10.1007/s00787-021-01832-4

**Published:** 2021-07-10

**Authors:** Kirsten R. Müller-Vahl, Natalia Szejko, Cara Verdellen, Veit Roessner, Pieter J. Hoekstra, Andreas Hartmann, Danielle C. Cath

**Affiliations:** 1 Clinic of Psychiatry, Social Psychiatry and Psychotherapy, Hannover Medical School, Carl-Neuberg-Str. 1, 30625 Hannover, Germany; 2 Department of Neurology, Medical University of Warsaw, Warsaw, Poland; 3 Department of Bioethics, Medical University of Warsaw, Warsaw, Poland; 4 Department of Neurology, Yale School of Medicine, Yale University, New Haven, USA; 5 PsyQ Nijmegen, Parnassia Group, Nijmegen, The Netherlands; 6 Department of Child and Adolescent Psychiatry, TU Dresden, Dresden, Germany; 7 Department of Child and Adolescent Psychiatry, University Medical Center Groningen, University of Groningen, Groningen, The Netherlands; 8 Department of Neurology, Hôpital de la Pitié-Salpêtrière, Paris, France; 9 Department of Specialist Trainings, GGZ Drenthe Mental Health Institution, Assen, The Netherlands; 10 Department of Psychiatry, University Medical Center Groningen, Rijks University Groningen, Groningen, The Netherlands; 11 TicXperts, Heteren, The Netherlands

**Keywords:** Tourette syndrome, Tics, Comorbidities, Guidelines, Treatment, Classification

## Abstract

In 2011 a working group of the European Society for the Study of Tourette syndrome (ESSTS) developed the first European Guidelines for Tourette syndrome (TS) published in the ECAP journal. After a decade ESSTS now presents updated guidelines, divided into four sections: Part I: assessment, Part II: psychological interventions, Part III: pharmacological treatment and Part IV: deep brain stimulation (DBS). In this paper, we summarise new developments described in the guidelines with respect to assessment and treatment of tics. Further, summary findings from a recent survey conducted amongst TS experts on these same topics are presented, as well as the first European patient representative statement on research. Finally, an updated decision tree is introduced providing a practical algorithm for the treatment of patients with TS. Interestingly, in the last decade there has been a significant shift in assessment and treatment of tics, with more emphasis on non-pharmacological treatments.

Tourette syndrome (TS)^1^ is a neurodevelopmental disorder at the crossroads between neurology, neuropaediatrics, and psychiatry. This is reflected for instance in the notion that tics, the hallmark of TS, are the result of involuntary motor disinhibition on the one hand, but are on the other hand, at least in part, under volitional control and can be voluntarily suppressed.

In 2011 ESSTS working groups have published the first “European Clinical Guidelines for Tourette syndrome and Other Tic Disorders” in the ECAP journal. Structured in four parts, these guidelines summarised the best available evidence combined with best practice expert consensus on the assessment and treatment of TS and related conditions: Part I: assessment [3], Part II: psychological interventions [17], Part III: pharmacological treatment [14], and Part IV: deep brain stimulation (DBS) [9]. The ESSTS guidelines have since then been used throughout Europe and have been cited over 500 times.

In the current special section of ECAP, we present an update of the four parts of the ESSTS guidelines published in 2011, supplemented with a decision tree providing a practical algorithm for the treatment of patients with TS (see Fig. 1) and a patient representative statement on research priorities. All parts together provide a comprehensive guideline that covers assessment and all forms of treatments. Since both empirical research findings and clinical knowledge are important elements of clinical guidelines, these guidelines therefore entail not only a thorough review of the evidence-based literature, but also the results of a survey conducted among ESSTS experts on the assessment and treatment of TS, which are incorporated in each part of the current guidelines.

**Fig. 1:**
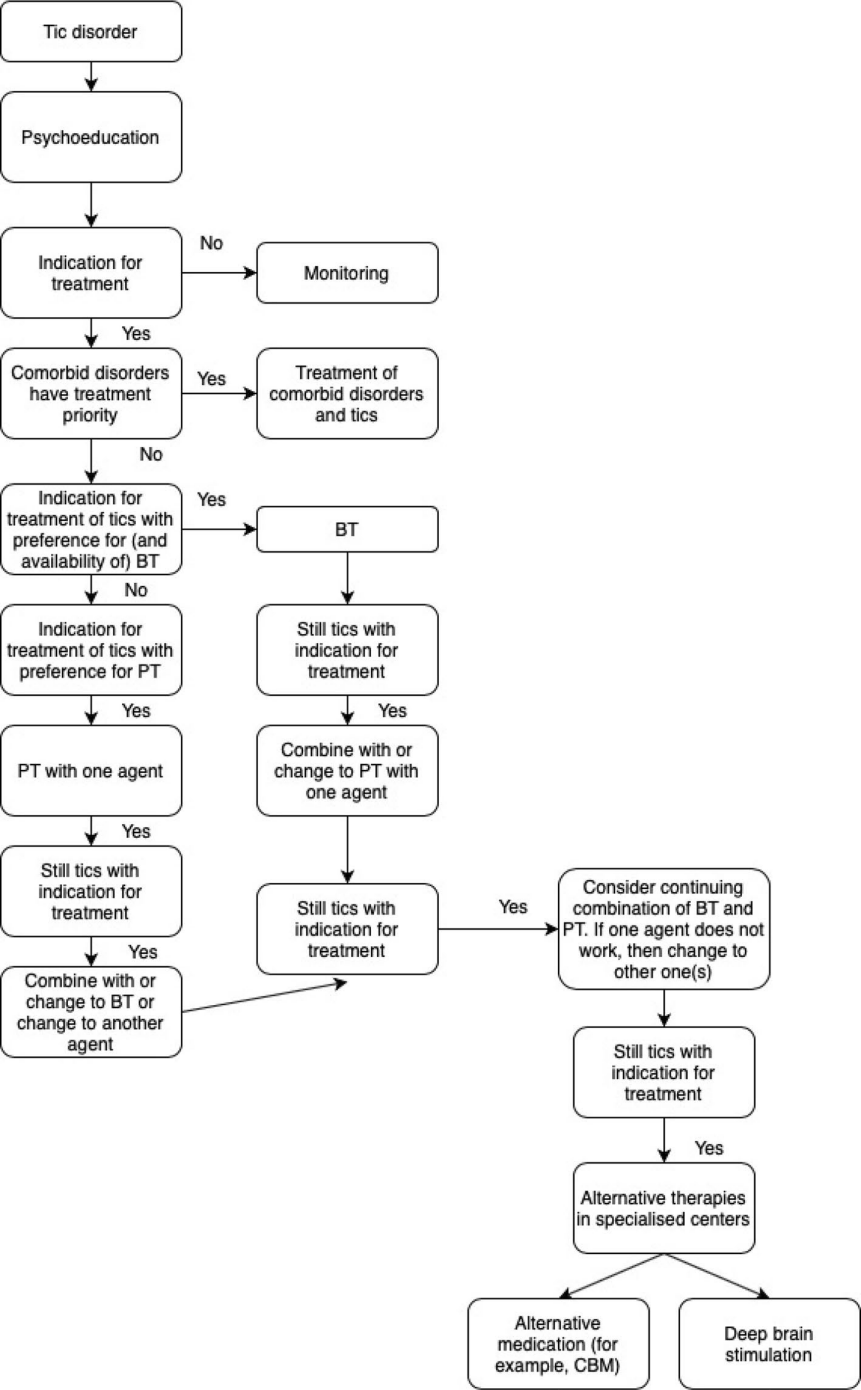
Algorithm for the treatment of patients with TS based on shared clinician patient decision making (adapted with permission from [14], Springer). *TS* Tourette syndrome, *PT* pharmacotherapy, *BT* behaviour therapy, *CBM* cannabis-based medicine

Recently the American Academy of Neurology (AAN) has published a systematic review [12] as well as practice guideline recommendations [13]—in which several ESSTS experts participated—on the effectiveness and safety of treatments for tics. Nonetheless, in our opinion, the present updated European clinical guidelines have a *raison d'être.* Through surveying ESSTS experts on assessment and treatment in both clinical practice and research, we were able to specifically incorporate knowledge and expertise from a large number of European experts into the guidelines. Further, despite overlap between Europe and the US/Canada, there are notable differences with respect to assessment and interventions in clinical practice. This is reflected by differences in health care use and organisation, in patient preferences and in first choice of pharmacological agents, availability and application of behavioural treatments, costs of treatment, and approval status. In the following paragraphs we summarise the most important conclusions formulated in each part of the guidelines.

In Part I: assessment, we have incorporated the newly implemented DSM-5 and ICD-11 criteria. We summarise available literature that includes newly developed tic rating scales and give concrete recommendations for assessments of tics and psychiatric comorbidities in the context of both routine practice and research. In addition, we advice how to differentiate tics and functional “tic-like” movements and “Tourette-like” behaviours [11]. In the DSM-5, the position of tic disorders has remained largely the same as in DSM-IV-TR, classifying TS as a “neurodevelopmental disorder”, alongside attention deficit/hyperactivity disorder (ADHD), intellectual disabilities and autism spectrum disorder (ASD). In the ICD-11, in contrast, tic disorders have been removed out of the ICD-10 category “Behavioural and emotional disorders with onset usually occurring in childhood and adolescence” and reassigned to the movement disorders section. In our opinion, this disregards the growing body of both genetic and clinical evidence that tic disorders are related to developmental and psychiatric disorders rather than to neurological disorders [2, 4]. Furthermore, in our opinion, there is no scientific evidence to support introduction of a subcategory “Infectious or post-infectious tics” (8A05.10) in the category “8A05.1 Secondary tics” [8]. Therefore, we do not recommend assessment of children for Paediatric Autoimmune Neuropsychiatric Disorders Associated with Streptococcal infections (PANDAS)-related TS. Moreover, introduction in ICD-11 of the secondary tics subcategory named “Tics associated with developmental disorders” (8A05.1.1) leaves room for confusion; when a person meets criteria for both tics and ASD or ADHD, he/she can be classified as suffering from either “Tics associated with developmental disorders” (8A05.11), or from TS in combination with ASD. In our opinion, current evidence indicates that tic disorders *are* by definition neurodevelopmental disorders.

With respect to behavioural interventions, in part II: psychological interventions, we outline substantial progress that has been made since 2011. Most importantly, since then several randomised controlled trials (RCTs) have been published on habit reversal treatment in both children and adults. As a result, behavioural treatment is currently regarded as the treatment of choice in reducing tics. Accordingly, with this up-date guidelines we changed the order of part II and part III. Further, internet-based modules of established behavioural treatments have been developed to render behaviour therapy more accessible. In addition, adaptations have been made to broaden the focus of behavioural treatment from reducing tic severity only to improving the individual’s overall quality of life.

With respect to pharmacological treatment (part III), most importantly, the antipsychotic agent aripiprazole has been proven effective in the treatment of tics in large-scale RCT [15, 19] and is currently the most frequently prescribed drug in Europe according to the ESSTS survey. During the last decade, three important trends can be noted. First, in contrast to the situation pre-2011, almost all RCTs have been sponsored by pharmaceutical companies which clearly increased the database but also bears the risk of bias [6]. Second, according to the AAN guidelines the traditional Chinese medicine products 5-ling granule and Ningdong granule have now made it to the list of compounds showing moderate confidence in evidence of treatment effects. However, we are not in accordance with this confidence for the following reasons: (i) Investigational Medicinal Product Dossier (IMPD) information that includes safety information of these agents is extremely limited; and (ii) agents contain products such as dried human placenta and therefore are not allowed on the European market. During recent years, several large scale RCTs have been conducted in China [14]. Although they may contain potentially important information, they have been almost exclusively published in Chinese [18]. Therefore, it is impossible to judge upon the quality of these trials for readers not mastering Chinese.

Although some new pharmacological compounds are currently under investigation to treat tics, first results were disappointing: both the vesicular monoamine transport (VMAT) inhibitor deutetrabenazine [16] as well as the inhibitor of the monoacylglycerol lipase (MAGL) and selective modulator of the endocannabinoid system Lu AG06466 (former ABX-1431) [10] did not meet the primary endpoint of tic reduction in phase 2/3 studies. Therefore, results from RCTs investigating efficacy and safety of ecopipam, a selective antagonist of dopamine D1-type receptors (ClinicalTrials.gov Identifier: NCT01244633), but also nabiximols, a cannabis extract containing tetrahydrocannabinol (THC) and cannabidiol (CBD) at a 1:1 ratio (ClinicalTrials.gov Identifier: NCT03087201), are highly anticipated.

With respect to deep brain stimulation (DBS), in part IV, we present increasing knowledge about efficacy in TS, although numbers and sample sizes of RCTs are still very limited. Reported effects are modest and partly even contradictory with effect sizes of tic improvement between 0.36 and 1.56. European contributions to a meta-analysis [1] and data of the International Tourette syndrome DBS Public Database and Registry [7] have contributed significantly to interpret the heterogeneous results [1, 7].

In order to incorporate clinical knowledge that reflects actual clinical practice in Europe into our revised and updated ESSTS guidelines, between October and November 2019 we conducted an online survey among ESSTS members. This was a follow-up to a prior survey carried out in the context of the 2011 guidelines, which allowed to capture the changes in assessment and treatment practices over the last decade [14]. We received responses from 59 experts from 17 different European countries predominantly from specialised outpatient clinics seeing on average 72 (range 0–600) children, 64 (range 0–666) adolescents, and 40 (range 0–300) adults with tics per year. Of note, experts encompassed child and adolescent psychiatrists (*n* = 20, 34%), psychologists (*n* = 11, 19%), adult neurologists (*n* = 13, 22%), and adult psychiatrists (*n* = 11, 19%) (several answers missing). Remarkably, 53% (*n* = 31) of experts conduct both clinical and research work, while 34% (*n* = 20) work only clinically and 10% (*n* = 6) are exclusively dedicated to research (several answers missing).

While detailed results of the survey have been added to the four different parts of the updated guidelines, here we briefly highlight the most relevant findings: (i) there is large agreement amongst European TS experts that in clinical practice tic severity assessment is based on clinical judgement complemented with observational and interview data including the Yale Global Tic Severity Scale (YGTSS) [5], the latter being the most widely used rating scale for tic assessment both in clinical practice and research; (ii) for the majority of ESSTS experts shared decision-making is common practice in the treatment of patients with TS, aiming to help patients to reach evidence-informed and value-congruent medical decisions; (iii) behavioural therapy was reported to be the first line treatment in both children and adults with tics, but was available in only 57% of centres. In contrast, 65% of experts consider pharmacotherapy when requested; (iv) all over Europe, there is still a substantial lack of trained psychotherapists so that only about half of the patients recommended for behavioural therapy can receive it; (v) as a consequence, internet (known as telemedicine or telehealth) as well as group-based treatment strategies are being introduced in clinical routine practice. Fortunately, different RCTs are currently ongoing investigating the efficacy of different kinds of internet-delivered behavioural therapy; (vi) the majority of experts recommends pharmacotherapy when behavioural therapy has been unsuccessful (72%) or in combination with behavioural therapy for severely affected patients (89%); (vii) compared to 2011, ESSTS experts have shifted from risperidone to mostly using aripiprazole as a first-line therapy followed by risperidone, clonidine, guanfacine (children) and topiramate (adults), respectively; (viii) remarkably, haloperidol, although being the only officially licenced drug for tic treatment in most European countries, is no longer used as a preferred drug by European experts (used by 6 (10%) experts in children/adolescents and 7 (12%) in adults, respectively); (ix) ESSTS experts consider DBS only in carefully selected and otherwise treatment-refractory patients corresponding to fewer than 3% of all patients. Although introduced already in 1999, DBS is still offered in only about 25% of highly specialised TS clinics.

Finally and uniquely, a paper by patients representatives in Europe who conducted a world-wide survey among patient advocacy groups, describes the patient perspective on different research topics. Unsurprisingly, three quarters of the 2000 respondents indicated that they would prefer research into the topic “how to treat TS and/or decrease symptoms”.

In conclusion, our revised ESSTS guidelines contain updated information on recent developments on assessment and treatment of TS, combined with a patient representative statement, which expresses the close and fruitful collaboration between advocacy groups and experts in the ESSTS community.

## Footnotes

1 We use the term TS in these guidelines, wherever information also applies to other forms of tic disorders. Only if there are substantial, well-known differences between TS and other forms of tic disorders, we use TS or other terms, e.g. transient or chronic motor tic disorder.
